# Postoperative IOP prophylaxis practice following uncomplicated cataract surgery: a UK-wide consultant survey

**DOI:** 10.1186/1471-2415-5-24

**Published:** 2005-10-07

**Authors:** Usha Zamvar, Baljean Dhillon

**Affiliations:** 1 Department of Ophthalmology, Stirling Royal Infirmary, Livilands, Stirling, UK; 2 Princess Alexandra Eye Pavilion, Chalmers Street, Edinburgh, UK

## Abstract

**Background:**

In order to minimise postoperative intraocular pressure (IOP) rise, after routine uncomplicated cataract surgery, prophylaxis may be adopted. Currently, there are no specific guidelines in this regard resulting in wide variation in practice across the UK. We sought to document these variations through a questionnaire survey.

**Methods:**

A questionnaire was sent to all consultant ophthalmic surgeons in the UK.

**Results:**

62.6% of surgeons did not use any IOP lowering agents. 37.4% surgeons routinely prescribed some form of medication. The majority (86.8%) used oral diamox. 20.6% of surgeons said they based their practice on evidence, 43.3% on personal experience, and 17.6% on unit policy. Surprisingly, among the two groups of surgeons (those who gave routine prophylaxis, and those who did not) the percentages of surgeons quoting personal experience, unit policy, or presence of evidence was strikingly similar. The timing of the first postoperative IOP check varied from the same day to beyond 2 weeks. Only 20.2% of surgeons had ever seen an adverse event related to IOP rise; this complication is thus very rare.

**Conclusion:**

This survey highlights a wide variation in the practice and postoperative management of phacoemulsification cataract surgery. What is very striking is that there is a similar proportion of surgeons in the diametrically opposite groups (those who give or do not give routine IOP lowering prophylaxis) who believe that there practice is evidence based. The merits of this study suggests that consideration must be given to drafting a uniform guideline in this area of practice.

## Background

Phacoemulsification and intra-ocular lens implantation (PhIOL) is one of the most cost-effective, elective surgical interventions. In order to minimise postoperative intraocular pressure (IOP) rise, prophylaxis may be adopted. Currently, there are no specific guidelines for prophylaxis in uncomplicated cataract surgery. We studied current prophylaxis practice in a UK-wide survey which showed wide variation in prophylaxis practice.

## Methods

We conducted a pilot, self-administered postal-based survey of the Scottish ophthalmic consultants. The results of this survey suggested variation in prophylaxis practice for IOP rise, duration until 1^st ^IOP monitoring, and management of elevated IOP following uncomplicated phacoemulsification with intraocular lens implantation with no coexisting comorbidity. This prompted us to extend our enquiry across the U.K. We obtained the names and addresses of ophthalmic consultants working across the United Kingdom, from the Royal College of Ophthalmologists, London. A questionnaire (Appendix 1) was sent to all the consultants between April and July 2003. The data were entered on a spreadsheet using Microsoft Excel. Data was analysed using SPSS 11(SPSS Inc, Chicago, IL).

## Results

The questionnaire was sent to 834 ophthalmic surgeons, and 515 (61.7%) responded. No reminders were sent. Ten did not perform cataract surgery and their responses were excluded from further analysis.

### Routine use of IOP prophylaxis following uncomplicated phacoemulsification and lens implant surgery

Of the 505 surgeons, who performed cataract surgery, 316 (62.6%) did not use any IOP lowering agent following uncomplicated phacoemulsification, and lens implant surgery. The remaining 189 (37.4%) routinely prescribed some form of medication for IOP prophylaxis.

Of the 189 surgeons who used some form of prophylaxis, oral diamox was used by 164 (86.8%), and a topical agent was used by 21 (11.1%). The remaining 4 (2.1%) surgeons used both.

Of the 168 surgeons who used oral diamox, 113 (67.2%), gave only one dose, 41 (24.4%) gave two doses, 9 (5.3%) gave three doses, and 5 (2.9%) gave four or more doses. Of the 25 surgeons who used topical agents, 22 (88%) gave only one dose, and 1 (4%) surgeon each gave 3, 5, and 6 doses.

### Basis of IOP prophylaxis practice

**Figure 1:**
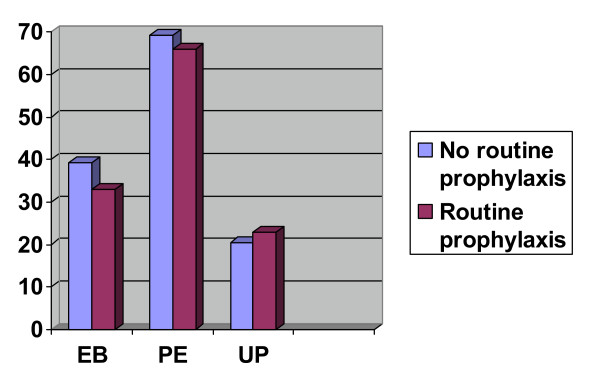
Basis of practice. EB: Evidence Based PE: Personal Experience UP: Unit Policy

We questioned the surgeons about the basis of their practice using a forced choice selection of a) based on evidence, b) personal experience, or c) as a matter of unit policy. Surgeons were allowed to select more than one option. Two hundred and ten (41.5%) surgeons did not reply, 104 (20.6%) said their practice was based on evidence, 219 (43.3%) said their practice was based on personal experience, and unit policy was the basis for 89 (17.6%). We analysed further the basis of practice, according to whether routine prophylaxis was used or not, and which drug was used. A total of 316 surgeons did not prescribe IOP lowering agent routinely. Of these, 209 surgeons (66.1%) chose not to reply when asked about the basis of their practice. Of the 107, who chose to reply, 42 (39.2%) said their practice was based on evidence, 74 (69.1%) said their practice was based on personal experience, and 22 (20.5%) on unit policy. One hundred and eighty nine surgeons routinely used some form of IOP lowering agent. Of these only one surgeon (0.5%) did not reply, to the question regarding the basis of their practice. Of the 188 who chose to reply, 62 (32.9%) said their practice was based on evidence, 124 (65.9%) said their practice was based on personal experience, and 43 (22.8%) on unit policy. The difference in the proportion of surgeons in the two groups choosing not to reply to this question is significant (p < 0.001). Among the respondents, the percentages of surgeons quoting personal experience, unit policy, or presence of evidence is strikingly similar (figure 1).

### Time of first postoperative IOP check

The timing of the first postoperative IOP check varied. Fifty-five surgeons (10.9%) reported the first IOP check was carried out on the same day, 150 (29.7%) on the first postoperative day, 105(20.8%) by the first week, 136 (26.9%) at 2 weeks, and 48 (9.5%) beyond 2 weeks. Nine surgeons (1.8%) said they never check IOP routinely. There was no significant difference in the timing of the first postoperative check between the users and non-users of IOP prophylaxis.

To assess whether the timing of postoperative IOP measurement has any impact on the incidence of reported adverse events, we looked at the following:

a) the various time points at which postoperative IOP measurements were made amongst the two groups of surgeons (those who give routine prophylaxis versus those who don't). (Table 1)

b) the relation between the timing of first postoperative IOP check and the reported experience with adverse events. (Table 2)

There was no statistically significant association between the timing of first IOP check, and the practice of giving routine prophylaxis. Similarly, there was no statistically significant association between the timing of first IOP check, and the reported experience with adverse events.

### Adverse event related to postoperative intraocular pressure elevation

One hundred and two surgeons (20.2%) had seen an adverse event related to IOP rise, compared to 396 (78.4%) who had not. 7 (1.4%) surgeons did not answer this question.

Of the 189 surgeons who routinely use IOP prophylaxis, 31 (16%) had encountered an adverse event in their practice, and 157 (83%) had not. Of the 316 surgeons who did not routinely use any prophylaxis, 71 (22%) had seen an adverse event, compared to 239 (75.6%) who had not. The difference was not significant (p = 0.10). A variety of adverse events were reported including corneal oedema, central retinal vein occlusion, ocular pain, optic neuropathy.

## Discussion

Cataract extraction is one of the most commonly performed and successful surgical procedures. This survey highlights a wide variation in the practice in the postoperative management of phacoemulsification cataract surgery.

Raised intraocular pressure is one of the most common complication following cataract surgery, requiring specific treatment [1-6]. Many treatment strategies of blunting acute post-op IOP spikes have been proposed, including use of prophylactic intracameral cholinergic agents and topical and systemic antiglaucoma medication [7-10]. However, this does not seem to eliminate spikes and many have found their effect to be negligible compared to a placebo [11-14]. The current literature on medical prophylaxis is conflicting [15-18] Most of the antiglaucoma agents used to prevent or lessen IOP increase postoperatively have limitations. 87% of responders who use IOP prophylaxis prefer oral Diamox over the topical agents. Oral Diamox or, Acetazolamide, a systemic sulphonamide inhibitor of carbonic anhydrase enzyme, reduces the flow of aqueous humor, thereby lowering the IOP. Acute urinary retention amongst men with prostatic enlargement and falls amongst the elderly may also occur with oral Diamox. Less serious side effects include thirst, drowsiness, polyuria and paraesthesia. This may result in accidents in elderly patients who have just undergone ocular surgery. More severe adverse reactions include fatal aplastic anaemia, sulfaallergy cross sensitivity and acid base disturbance [21].

Iopidine and Timoptol are the most common topical agents used for post-op IOP prophylaxis as shown in the survey. A number of clinical trials studying the effect of pre and post-operative use of Apraclonidine and Timoptol in reducing post-op rise have shown variable results[8,9,12,14,17,18].

Our survey also demonstrates a wide variation in the timing of the first IOP check. Only 10.9 % of our responders check IOP on the day of surgery. These patients do not visit the hospital on the first postoperative day, which is very convenient for them, and for overall majority of patients, visual outcome is not compromised when routine next day review is omitted after phacoemulsification surgery [19,20].

This reflects the relatively low frequency of severe IOP elevation one day postoperatively, the self limiting nature of IOP spikes and the tolerance of a healthy eye.

This survey also showed that only 20.2% surgeons had ever encountered an adverse event related to IOP rise. The vast majority of surgeons (78.4%) had never encountered one. An adverse event related to IOP rise is rare [20]. Assuming that each surgeon performs 400 cataract operations per year, and that the 20.2% surgeons who had encountered the complications see it twice a year, the incidence of this complication would be 0.1%. When the incidence of any complication is this low, it may be difficult to organise a randomised controlled trial to show if the use of IOP lowering prophylaxis is effective or not, as the sample size would run in tens of thousands. For example, in this case, we would need 700,000 patients to be randomised for a study with a power of 80%, to show a difference of 20% in the incidence of adverse event related to IOP rise. This is clearly an impossible task. Randomised controlled trials have been conducted to assess the role of IOP prophylaxis [7-10,12-18], but they have used the IOP level as a surrogate marker. There may be fluctuations in the IOP levels, and a statistically significant difference between the two groups, but whether this actually results in a change in the incidence of an adverse event related to IOP rise is unproven.

It was a weakness of our questionnaire that we did not ask the surgeons about their annual cataract surgical volumes, and the number of complications they had encountered. Another point of this study that we would like to highlight is that a high proportion of surgeons not prescribing routine IOP prophylaxis chose not to give a reason for the basis of their practice.

## Conclusion

In summary, this survey shows a very wide variation in practice regarding postoperative management of patients undergoing phacoemulsification with intraocular lens implant. What is very striking is that there is a similar proportion of surgeons in the diametrically opposite groups (those who give or don't give routine IOP lowering prophylaxis) who believe that their practice is evidence based. Personal experience was cited by a large percentage of surgeons in each group. Practice of medicine is not necessarily evidence based. Reasons include [22] clinical experience, over-reliance on surrogate outcomes, ritual and mystique. Our survey adds another explanation: interpretation of the evidence in different ways, perhaps to fit with one's clinical practice.

Whilst the authors would not wish to be prescriptive in post PhIOL prophylaxis, the merits of this study suggests further consideration might be given to drafting a uniform guideline in this area of practice.

Appendix 1:

1) Do you routinely give any intraocular pressure lowering agent to your patients (**without co-morbidities**) following **uncomplicated**, phacoemulsification and lens implant surgery?

a) Yes

b) No

2) If Yes, which one of the following?

a) Topical medication (Please specify the name of the drug)

b) Oral Diamox- 250 mg/500 mg

3) At what stage following the surgery, do the patients receive the above medication?

a) at the end of the surgical procedure

b) in the recovery area

c) on the ward

d) at home (after discharge from hospital)

4) How many doses are given?

a) one

b) two

c) three

5) What is the basis of your practice? (you may tick more then one)

a) Based on evidence

b) Based on personal experience

c) Unit policy

6) Do you give subconjunctival injection at the end of the procedure?

a) yes

b) no

7) If yes, which of the following ?

a) subconj antibiotic

b) subconj steroid

c) both

8) When do the patients have their intraocular pressure checked for the first time after surgery?

a) Few hours after surgery

b) First postoperative day

c) 1 week postop

d) 2 weeks postop

9) Have you come across a sight threatening condition caused by raised postoperative IOP?

a) Yes (please specify)

b) No

10) How would you treat a patient with significantly raised IOP (> 30 mm Hg) within the first 24 hours following surgery?

a) paracentesis through the 2^nd ^port

b) oral diamox

c) I/V diamox

11) What post-op medication do you give to your patients and for how long?

## Competing interests

The author(s) declare that they have no competing interests.

## Authors' contributions

Details of contributors and guarantor:

BD had the original idea, UZ and BD drafted the questionnaire, UZ tabulated the replies, performed the statistical analysis and wrote the original manuscript, UZ and BD contributed to the final manuscript. BD is the guarantor.

## Pre-publication history

The pre-publication history for this paper can be accessed here:



## Figures and Tables

**Table 1:** 

Timing of first IOP check	Surgeons give routine prophylaxis (n = 189)	Surgeons do not give routine prophylaxis (n = 316)
Same Day	22 (11.6%)	33 (10.4%)
1^st ^postop day	52 (27.5%)	98 (31.0%)
By 1^st ^week	35 (18.5%)	70 (22.1%)
At 2 weeks	59 (31.2%)	77 (24.3%)
Beyond 2 weeks	17 (8.9%)	31 (9.8%)
Never	4 (2.1%)	5 (1.5%)
No reply	0 (0%)	2 (0.6%)

Legend: This table shows the timing of the first postoperative IOP check in the two groups of surgeons (those who routinely give or don't give prophylaxis). There is no statistical significant difference between the two groups.

**Table 2:** 

Timing of first postop IOP check	No. of surgeons (n = 503)	Surgeons who have experienced adverse events
Same day	55 (10.9%)	15 (27.2%)
1^st ^post-op day	150 (29.7%)	25 (16.6%)
By first week	105 (20.8%)	20 (19.0%)
At 2 weeks	136 (26.9%)	36 (26.4%)
Beyond 2 weeks	48 (9.5%)	6 (12.5%)
Never	9 (1.8%)	0 (0%)

Legend: Relation between timing of first IOP check and reported experience with adverse events. There is no significant trend noticed.

## References

[B1] Tan JH, Newman DK, Klunker C, Watts SE, Burton RL (2000). Phacoemulsification cataract surgery: is routine review necessary on the first postoperative day?. Eye.

[B2] Tufail A, Foss AJ, Hamilton AM (1995). Is the first day postoperative review necessary after cataract extraction?. Br J Ophthalmol.

[B3] Whitefield L, Crowston J, Little BC (1996). First day follow up for routine phacoemulsification?. Br J Ophthalmol.

[B4] Cohen VM, Demetria H, Jordan K, Lamb RJ, Vivian AJ (1998). First day postoperative review following uncomplicated phacoemulsification. Eye.

[B5] Dinakara S, Desai SP, Raj PS (2000). Is the first postoperative day review necessary following uncomplicated phacoemulsification surgery?. Eye.

[B6] Desai P, Minassian DC, Reidy A (1999). National cataract surgery survey 1997–98: a report of the results of the clinical outcomes. Br J Ophthalmol.

[B7] Zohdy GA, Rogers ZA, Lukaris A, Sells M, Roberts-Harry TJ (1998). A comparison of the effectiveness of dorzolamide and acetazolamide in preventing postoperative intraocular pressure rise following phacoemulsification surgery. J R Coll Surg Edinb.

[B8] Araie M, Ishi K (1993). Effects of apraclonidine on intraocular pressure and blood-aqueous barrier permeability after phacoemulsification and intraocular lens implantation. Am J Ophthalmol.

[B9] Fry LL (1992). Comparison of the postoperative intraocular pressure with Betagan Betoptic, Timoptic, Iopidine, Diamox, Pilopine Gel, and Miostat. J Cataract Refract Surg.

[B10] Scherer WJ, Mielke DL, Tidwell PE, Hauber FA (1998). Effect of Latanoprost on intraocular pressure following cataract extraction. J Cataract Refract Surg.

[B11] Barak A, Desatnik H, Ma-Naim T, Ashkenazi I, Neufeld A, Melamed S (1996). Early postoperative intraocular pressure pattern in glaucomatous and non glaucomatous patients. J Cataract Refract Surg.

[B12] Bomer TG, Lagreze WD, Funk J (1995). Intraocular pressure rise after phacoemulsification with posterior chamber lens implantation: effect of prophylactic medication, wound closure, and surgeons' experience. Br J Ophthalmol.

[B13] Rainer G, Menapace R, Schmetterer K, Findl O, Georgopoulos M, Vass C (1999). Effect of Dorzolamide and latanoprost on intraocular pressure after small incision cataract surgery. J Cataract Refract Surg.

[B14] Sterk CC, Renzenbrink-Bubberman AC, van Best JA (1998). The effect of 1% apraclonidine on intraocular pressure after cataract surgery. Ophthalmic Surg Lasers.

[B15] Ruiz RS, Wilson CA, Musgrove KH, Prager TC (1987). Management of intraocular pressure after cataract extraction. Am J Ophthalmol.

[B16] Byrd S, Singh K (1998). Medical control of intraocular pressure after cataract surgery. J Cataract Refract Surg.

[B17] Lai JS, Chua JK, Leung AT, Lam DS (2000). Latanoprost versus timolol gel to prevent ocular hypertension after phacoemulsification and intraocular lens implantation. J Cataract Refract Surg.

[B18] Duperre J, Grenier B, Lemire J, Mihalovits H, Sebag M, Lambert J (1994). Effect of timolol vs acetazolamide on sodium hyaluronate-induced rise in intraocular pressure after cataract surgery. Can J Ophthalmol.

[B19] Tinley CG, Frost A, Hakin KN, McDermott W, Ewings P (2003). Is visual outcome compromised when next day review is omitted after phacoemulsification surgery? A randomised controlled trial. Br J Ophthalmol.

[B20] Thirumalai B, Baranyovits PR (2003). Intraocular pressure changes and the implications on patient review after phacoemulsification. J Cataract Refract Surg.

[B21] Fraunfelder FT, Meyer SM, Bagby GC, Dreis MW (1985). Hematologic reactions to carbonic anhydrase inhibitors. Am J Ophthalmol.

[B22] Doust J, Del Mar C (2004). Why do doctors use treatments that do not work?. BMJ.

